# A Comparative Study of Selected Trace Element Content in Malay and Chinese Traditional Herbal Medicine (THM) Using an Inductively Coupled Plasma-Mass Spectrometer (ICP-MS)

**DOI:** 10.3390/ijms14023078

**Published:** 2013-02-01

**Authors:** Fairuz Liyana Mohd Rasdi, Nor Kartini Abu Bakar, Sharifah Mohamad

**Affiliations:** Department of Chemistry, Faculty of Science, University of Malaya, 50603 Lembah Pantai, Kuala Lumpur, Malaysia; E-Mails: kartini@um.edu.my (N.K.A.B.); sharifahm@um.edu.my (S.M.)

**Keywords:** trace elements, ICP-MS, traditional herbal medicines, method validation, microwave digestion

## Abstract

A total of 60 products of traditional herbal medicine (THM) in various dosage forms of herbal preparation were analyzed to determine selected trace elements (*i.e.*, Zn, Mn, Cu, Cd, and Se) using ICP-MS. Thirty types of both Chinese and Malay THMs were chosen to represent each population. The closed vessel acid microwave digestion method, using CEM MARS 5, was employed for the extraction of the selected trace elements. The digestion method applied was validated by using certified reference material from the Trace Element in Spinach Leaves (SRM1570a). The recoveries of all elements were found to be in the range of 85.3%–98.9%. The results indicated that Zn, Mn, Cu, Cd and Se have their own trends of concentrations in all samples studied. The daily intake concentrations of the elements were in the following order: Mn > Zn > Cu > Se > Cd. Concentrations of all five elements were found to be dominant in Chinese THMs. The essentiality of the selected trace elements was also assessed, based on the recommended daily allowance (RDA), adequate intake (AI) and the United States Pharmacopeia (USP) for trace elements as reference. The concentrations of all elements studied were below the RDA, AI and USP values, which fall within the essential concentration range, except for cadmium.

## 1. Introduction

The term “trace element” is applied to a group that is extremely small in quantity and plays a vital part in the metabolism of plants and animals [1]. In the human body, the trace element is defined as that which makes up 0.01% of the body’s mass [2]. There are a maximum of 25 trace elements distributed in multiple sites of a skeleton and in the iliac crest of 69 ancient human skeletons [3]. Each element has made a different contribution in order to make the human body function. Essential trace elements are needed for optimal function of the mammalian organism, for growth, healing and the activity of many metabolic processes [2].

Trace elements have both a curative and a preventative role in combating diseases. Trace elements, for example the metals selenium, zinc and copper, are essential to maintain the metabolism of the human body. However, non-essential metals such as cadmium and chromium lead to adverse effects, even though they are only present in trace amounts. Elements, in one form or another play an important role in the field of medicine, including the trace elements present in traditional herbal medicines (THM). The consumption of THM contributes to the intake of both essential and non-essential trace elements by the human body [4].

The World Health Organization (WHO) has estimated that around 65%–80% of the world population, especially in developing countries, depends essentially on plants for their primary healthcare [5]. THM use has been steadily rising with almost 70%–95% of citizens in major developing countries using THM for their primary health care needs [6]. Use of THM is quite convincing, since it is affordable for all people regardless of their income [7]. The WHO has defined herbal medicine as “herbs, herbal materials, herbal preparations and finished herbal products that contain active ingredients obtained from parts of plants, or plant materials, or combinations thereof” used to treat ailments [8–10] throughout the world [11].

THM has gained people’s satisfaction with its therapeutic outcomes [12,13] and there are perceptions that herbal medicines are inherently safe [14]. Furthermore, the dissatisfaction of patients towards orthodox medicine in terms of effectiveness and safety has also induced the use of THM [15]. According to Basgel and Erdemoglu, the therapeutic agents in herbal medicines are standardized herbal preparations consisting of complex mixtures of one or more plants, which are used in most countries for the management of various diseases [16].

Despite the claimed and wide belief in THM as being beneficial, there have been reports of acute and chronic intoxications resulting from its use [17–23]. Abbot *et al.* reported that not all products that are related to THM are free from adverse effects [24]. According to Mazzanti *et al.*, the adverse effects can be divided into two; intrinsic and extrinsic [25]. The intrinsic adverse effects are due to predictable toxicity, overdose, pharmacological interactions, idiosyncratic reactions (*i.e.*, allergy and anaphylaxis) and delayed effect (*i.e.*, carcinogenicity and teratogenicity). The quality of THM is affected by substitution, adulteration, contamination, misidentification, lack of standardization and inappropriate labeling, which belong to the extrinsic adverse effect group.

While many investigations of the quality of THM have been reported in the current literature [26], there is less emphasis on the trace element content of THM products [27]. The main reason for trace element monitoring was due to an increase in contamination of the general environment [28,29]. As a result of contamination, serious health hazards have occurred such as renal failure, symptoms of chronic toxicity and liver damage [30,31].

However, there have also been health hazard cases reported on THM *i.e.*, Ayurvedic herbal medicine products (AHMP) that use trace elements as their constituents [32]. Some consumers took AHMP containing lead (Pb) and were reported to demonstrate epilepticus [33], fatal infant encephalopathy [34], congenital paralysis and sensorineural deafness [35], and developmental delay [36]. Moreover, at least 55 cases of trace element intoxication associated with AHMP in adults and children have been reported in the United States and worldwide since 1978 [33–45].

As reported by Shaw *et al.*, there are 785 cases that have been evaluated with side effects related to THM. The cases reported were referred to as liver problems resulting from the use of Chinese THM for skin disorders and heavy metal poisoning caused by consuming Indian subcontinent remedies [31]. Garvey *et al.* also reported that the level of arsenic (As), lead (Pb) and mercury (Hg) in Asian remedies ranges from toxic (49% of the total number of samples analyzed) to those exceeding the public health guidelines for illness prevention (74%) even though they followed the instructions given on the packages [19].

However, with THM the essential parts still have to be taken into consideration. The experimental work done by Ajasa *et al.* does support the fact that THMs also contains nutrients and are rich in iron (Fe), phosphorus (P), magnesium (Mg), calcium (Ca), sodium (Na) and potassium (K) [4]. Other reports revealed that the trace elements present in THM display beneficial medicinal and therapeutic properties [46,47]. Obi *et al.* reported that heavy metal poisoning has decreased due to an improvement in industrial hygiene and environmental controls [48]. The trace element content in THMs should be monitored in order to prevent the consumer from suffering undesired effects. It is crucial to make sure that the trace element content is below the required limit for illness prevention. The dietary intakes for the bio-important trace elements have to be regulated to avoid poisoning and toxicity in the body [49–51].

The aim of this study was to determine the concentration of Zn, Mn, Cu, Cd, and Se in both Chinese and Malay THM. The work is a comparison of the trace element content between Chinese and Malay THM samples. The daily intake of all elements studied in both THM samples was compared with the recommended daily allowance (RDA), adequate intake (AI) and U.S. Pharmacopoeia (USP) for trace elements. Based on the daily intake of the selected trace element, their essentiality is classified as essential, non essential or non effective concentrations.

## 2. Results and Discussion

### 2.1. Method Validation

A microwave digestion program was found to be a capable method to be used for sample preparation of traditional herbal medicines thereby, taking into account information obtained when comparing recovery, the total time taken for analysis, operational difficulties, the amount of acid used and the safety requirements during the process. Table 1 and Table 2 show the results obtained for Zn, Mn, Cu, Cd, and Se in the analysis of SRM 1570A. Seven replicates of measurement (*n* = 7) were performed using microwave assisted digestion. During the ICP-MS analysis, a blank and six standard solutions of all elements were prepared in the range of 0–250 μg L^−1^ for all calibration curves. All plotted calibration curves showed that the linearity was almost perfect with a correlation coefficient of *r*^2^ = 0.9999. The accuracy study showed that the recoveries from the microwave assisted digestion method ranged from 85.3% to 98.9% as shown in Table 1. The precision of the method was achieved with the coefficient of variation (CV) for intraday and interday precision of <5% as shown in Table 2.

A total of 10 replicates (*n* = 10) of blank samples were analyzed and the mean values and their standard deviations were determined. Table 3 shows that the limit of detection (LOD) and limit of quantification (LOQ) were in the range of 0.03–0.38 ng L^−1^ and 0.10–1.15 ng L^−1^; respectively. The analytical method adopted was very sensitive towards all analytes studied.

### 2.2. Daily Intake Estimation

The daily intakes of Zn, Mn, Cu, Cd, and Se of each THM were obtained by multiplying the daily intakes of each product by the concentration of each element determined by ICP-MS. Then, the trace element intake was compared with the tolerable daily intakes for each element obtained from RDA, AI and USP.

### 2.3. Essentiality of Trace Elements Based on Their Estimated Daily Intake

The present study was apparently the first comparative study of Zn, Mn, Cu, Cd, and Se contents in both Chinese and Malay THM retailed in Malaysia. In this work, the essentiality of Zn, Mn, Cu, Cd, and Se content in Chinese and Malay THM products is discussed and the estimated daily intake provided by manufacturers is also highlighted.

**Figure 1:**
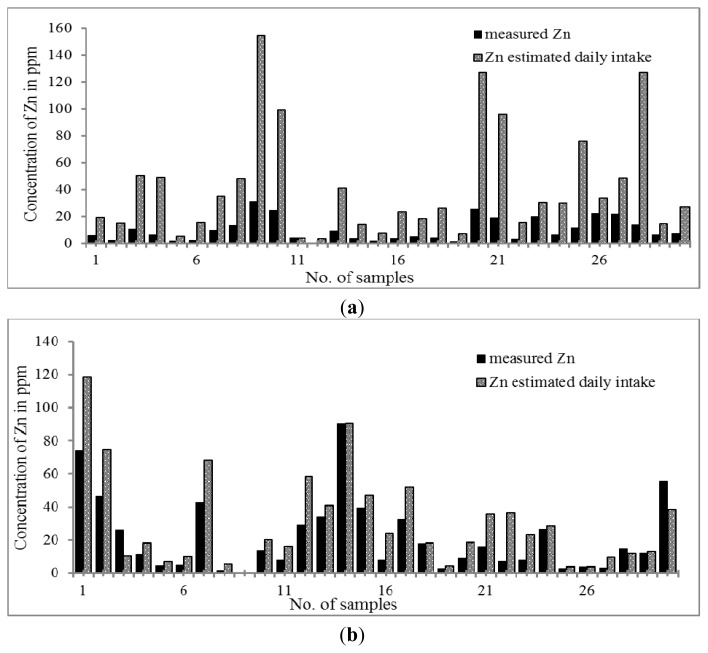
(**a**) Distribution of Zn content in Chinese THM with estimated daily intake; (**b**) Distribution of Zn content in Malay THM with estimated daily intake.

The trend for the distribution of Zn is shown in Figure 1a,b. The highest concentration of Zn was found in both Chinese and Malay THM samples. The results indicated that all Chinese and Malay THM samples contained Zn. The concentration of Zn was in the range of 0.61–30.90 μg g^−1^ and 0.29–74.03 μg g^−1^ in Chinese and Malay THM, respectively. Zn content was significantly higher in Malay THMs than in that of Chinese THMs. Based on the daily dosage of Zn, the percentage increment in concentration of Zn is higher in Chinese THMs by 3–9 fold while Malay THMs only show 1–3 fold. Nevertheless, the results indicate that the daily dose of Zn in Chinese and Malay THM was relatively lower compared to the recommended daily allowance (RDA) (2–12 mg per day). Previous studies proved that Zn is essential to the human body at the optimum level for reducing morbidity resulting from respiratory and diarrheal illnesses [52–55]. Furthermore, Zn deficiency can lead to growth retardation, immune dysfunctions and cognitive impairment [56].

**Figure 2:**
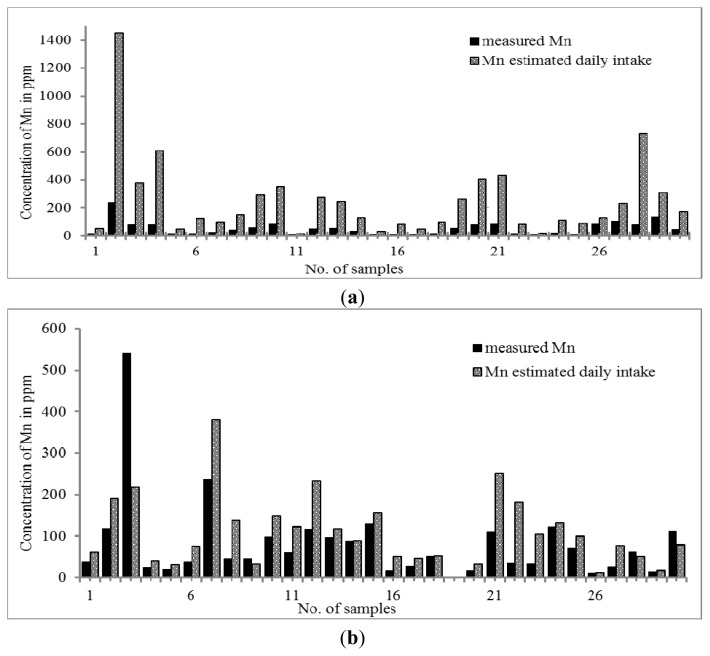
(**a**) Distribution of Mn content in Chinese THM with estimated daily intake; (**b**) Distribution of Mn content in Malay THM with estimated daily intake.

The distribution trend of Mn was similar to that of Zn. The results in Figure 2a,b showed that the concentration of Mn was slightly higher in Malay THM (0.31–542.10 μg g^−1^) than that of Chinese THM (10.21–241.88 μg g^−1^). When the daily intake of THM was taken into account, the concentration of Mn in Chinese THM was increased by up to 7.5 times but all Malay THMs only showed the increment of Mn in the range of 0.4–3.0 times, except for sample 22 (S22) with an increment of five times. Since there is no RDA value set for Mn, the adequate intake (AI) value was used as the reference. The calculated intake of both THMs revealed that the Mn content is an essential element although its concentration is within the value stated by adequate intake (AI) (0.003 to 2.6 mg per day). Aschner *et al.*, 2005, state that Mn is required in normal amino acid, lipid, protein and carbohydrate metabolism at essential concentrations. Nevertheless, high consumption of Mn can cause manganism; one of the neurodegenerative disorders that is due to the susceptibility of the brain towards excess of Mn [57].

**Figure 3:**
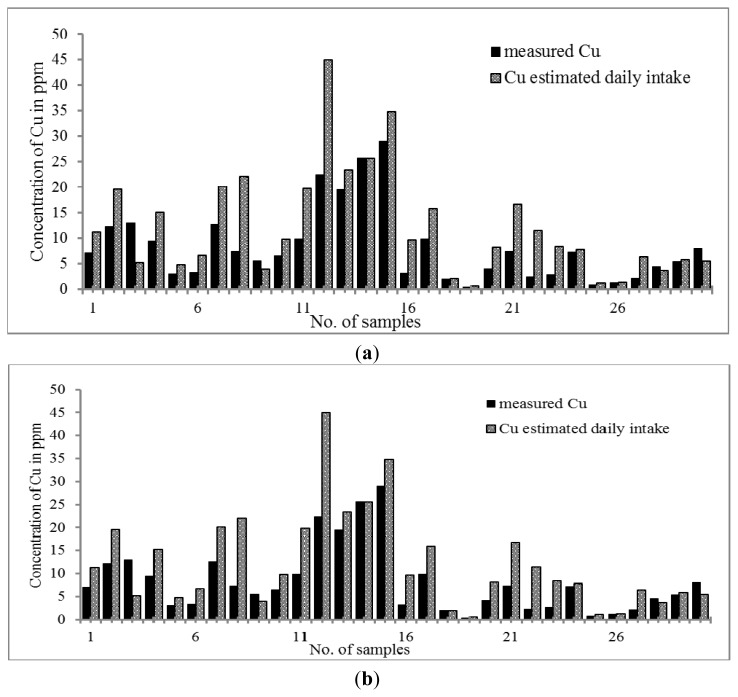
(**a**) Distribution of Cu content in Chinese THM with estimated daily intake; (**b**) Distribution of Cu content in Malay THM with estimated daily intake.

Figure 3a,b reveal that both Malay and Chinese THMs contain Cu in the range of 0.40–28.95 μg g^−1^ and 0.24–8.49 μg g^−1^; respectively. The pattern of distribution was quite similar to that of Mn and Zn. The Cu content was higher in Malay than Chinese THM whereas the daily intake of copper was higher in Chinese THM. The daily intake exhibits a trend wherein Chinese THM shows a higher Cu content. As far as both kinds of THM are concerned, there are significant increments of Cu. The concentration of Cu in Chinese THM increased from 0.24–8.49 μg g^−1^ to 0.80–33.95 μg day^−1^ while in Malay THM from 0.40–28.95 μg g^−1^ to 0.60–44.96 μg day^−1^. Cu contents were comparatively lower than the RDA (0.2 to 1.3 mg per day) value. However, the concentration of Cu present in both THMs was considerably essential. Previous studies showed that Cu plays an important role in the expression of certain human genes at the optimum concentration [58]. However, high consumption of Cu l causes chronic damage to the liver [59].

**Figure 4:**
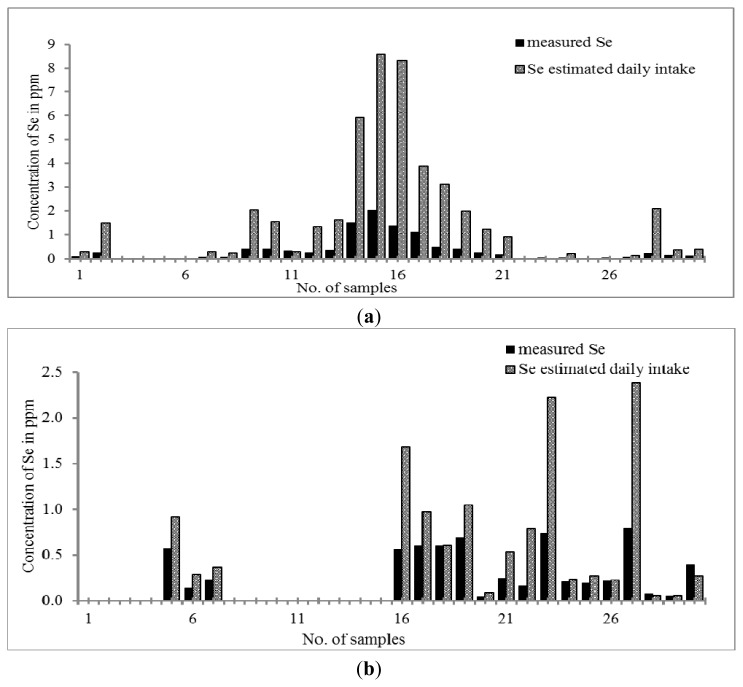
(**a**) Distribution of Se content in Chinese THM with estimated daily intake; (**b**) Distribution of Se content in Malay THM with estimated daily intake.

As can be seen in Figure 4a,b, only 80% of Chinese THM and 60% of Malay THM contain Se. The concentration of Se in Chinese THM is in the range of 0.03–2.03 μg g^−1^ which exhibits a higher concentration of Se than that of Malay THM samples (*i.e.*, 0.04–0.74 μg g^−1^). Only four out of thirty samples of Chinese THM contain Se greater than 2 μg L^−1^ (S14, S15, S16 and S17). THM contains different ingredients for different purposes. Surprisingly, none of Malay THM studied contains Se greater than 2 μg g^−1^. The calculated daily Se intake shows that the Se content of some of the Chinese THM samples was approaching the essential value recommended by RDA (20 to 55 μg per day) that is 0.23 to 8.85 μg day^−1^. The concentration of Se found in Chinese THM was found as significantly essential to the human body with the assumption that the consumer takes more than one type of THM per day. The prevalence study reported that the optimum concentration of Se in the blood would prevent oxidative damage, hence preventing people from atherosclerosis [60]. Recently, there are several studies that show that inorganic Se can enhance insulin activity by mediating insulin-like actions [61,62].

**Figure 5:**
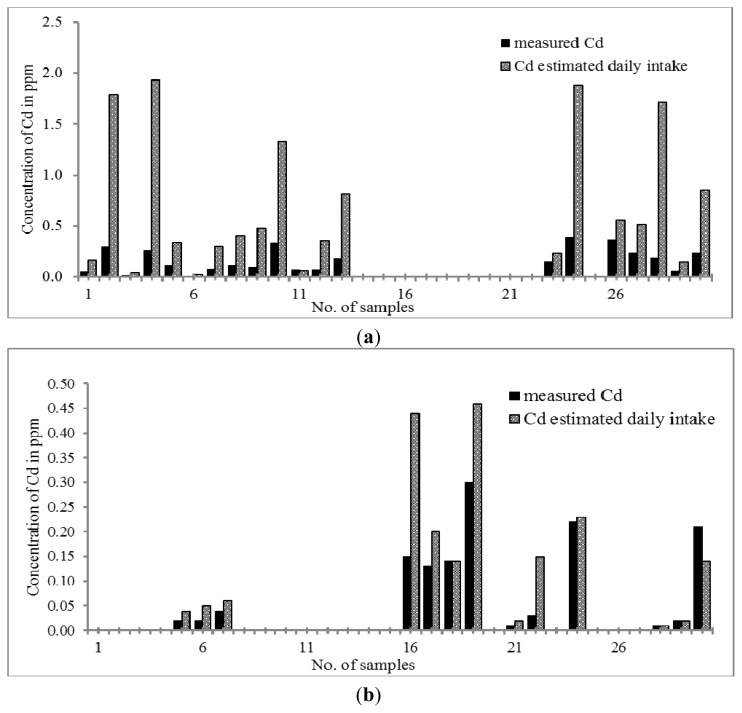
(**a**) Distribution of Cd content in Chinese THM with estimated daily intake; (**b**) Distribution of Cd content in Malay THM with estimated daily intake.

Cd is considered a toxic element. A previous finding showed that Cd could be toxic to placenta [63] due to its prohibition on cell proliferations [64–66]. By referring to Figure 5a,b, the concentration of Cd ranges between 0.07 and 0.39 μg g^−1^ and between 0.01 and 0.30 μg g^−1^ in Chinese THM and Malay THM; respectively. Both THMs were considered to have a low Cd content compared to the limit stated by the U.S. Pharmacopoeia (3 mg g^−1^). Only 63% of Chinese THM and 43% of Malay THM studied contains Cd. When the daily intake of both THMs was considered, the concentration of Cd in Chinese and Malay THM increased up to 0.03–1.79 μg day^−1^ and 0.01–0.44 μg day^−1^, respectively. Therefore, we can conclude that the concentrations of Cd in the daily intake of Chinese THM samples are still in the acceptance range and not approaching the maximum limit of cadmium stated by the U.S. Pharmacopoeia (USP). However, continuous consumption of Chinese THM will cause a huge accumulation of Cd in the body. Cd can cause intracellular damage such as protein denaturation, lipid peroxidation, generation of reactive oxygen species and DNA strand breaks [67–69].

The different formulation sizes were found to be one of the contributors towards the increment of concentration of trace elements in the THM samples. As the surface area of the formulation increases, the concentrations of trace elements present in both THMs also rises. The other likely contributor is the quantity of the formulation consumed daily. Some of the Chinese THM samples were prescribed to be taken in a very high quantity every day. At the same time, the concentrations of all elements studied increased with the increase of the daily formulation intake. The concentrations of all elements studied in Malay THM also showed a steady increment with daily intake. The types of capsules, the source of herbs and the manufacturing process play a significant role as well on the presence of trace elements in both Chinese and Malay THMs.

### 2.4. Statistical Analysis

Analysis of variance (one-way ANOVA) was used to test hypothesis about differences between element contents. In one-way ANOVA, no statistical difference at 95% confidence interval was observed between manganese, cadmium and selenium concentrations in Chinese and Malay THM populations except for zinc and copper. We noticed significant differences at 95% confidence interval in Mn, Zn, Cu, Se and Cd concentrations within each population.

## 3. Experimental Section

### 3.1. Sampling

Thirty samples of Malay and Chinese THMs were randomly purchased from herbal medical stores, kiosks, herbal medical dealers and local supermarkets in Kuala Lumpur, regardless of their uses. The formulations of THMs were found to be in several forms such as pellets, capsules, tablets, powders and “gummy pastes”. All the samples chosen were registered with the Malaysian Health Ministry Malaysia through the National Pharmaceutical Control Bureau.

### 3.2. Sample Preparations

The THM samples were directly used in analysis without prior treatment to avoid alteration of the concentration of trace elements in the samples. The concentrations of selenium, copper, manganese, zinc and cadmium in both Malay and Chinese THM samples were determined using an Agilent 7500a ICP-MS series (Agilent Technologies). A microwave digestion technique was applied for wet digestion of the THM samples. The National Institute of Standards and Technology (NIST) solid standard reference material (SRM) 1570a (Trace elements in Spinach) containing a concentration of specified trace elements served as positive and negative control to ensure the quality of the measurements.

### 3.3. Reagents and Materials

All reagents were of analytical reagent grade unless otherwise stated. Nitric acid (65% HNO_3_, suprapur Merck, Darmstadt, Germany) and hydrogen peroxide (30% H_2_O_2_, MOS J. T. Baker, Centre Valley, PA, USA) were used as a mixture in a ratio of 8 to 2 (HNO_3_:H_2_O_2_*v*/*v*). Ultrapure water (Milli-Q water purification system, Millipore, Billerica, MA, USA) with resistivity of 18.2 Ω cm^−1^ was used throughout the experiments including for all dilutions and for rinsing the vessels for microwave digestion. The plastic containers and glassware were cleaned by soaking in dilute HNO_3_ (1 + 9) and were rinsed with ultrapure water prior to use. All standards, reagent solutions and sample were kept in plastic containers and stored in the refrigerator before the measurements. A multi-element standard solution IV for ICP-MS (Fluka, Switzerland) was used to prepare the series of standard solutions of zinc (Zn), manganese (Mn), copper (Cu), cadmium (Cd), and selenium (Se). The concentration of Zn, Mn, Cu, Cd, and Se were 100, 20, 10, 10, and 100 μg L^−1^, respectively.

### 3.4. Preparation of Standard Solution

The multistandard solution was diluted with 5% nitric acid step by step; the concentration of Se and Zn were prepared by the gradient of 0.00, 25.00, 75.00, 125.00, 187.50 and 250.00 μg L^−1^, the concentration of Cd and Mn were prepared by the gradient of 0.00, 2.50, 7.50, 12.50, 18.75, 25.00 μg L^−1^, and the concentration of Cu was prepared by the gradient of 0.00, 5.00, 15.00, 25.00, 37.50, 50.00 μg L^−1^. The working standard solution was freshly prepared daily prior to the analyses.

### 3.5. Microwave Digestion

About 0.2 g of replicate sample of SRM 1570a was weighed using a Teflon vessel, and then a mixture of 8 mL of concentrated HNO_3_ and 2 mL of H_2_O_2_ was added. The digestion vessel was closed and heated in the CEM MARS microwave oven based on the parameters shown in Table 4. The obtained solutions were allowed to cool at room temperature, and then were filtered by Whatman No. 1 (110 mm pores size) filter paper into a 25 mL polypropylene (PP) volumetric flask. Acidified double distilled deionized water (with 5% *v*/*v* HNO_3_) was prepared and used for all dilutions throughout this work.

### 3.6. Instrumentation

An inductively coupled plasma-mass spectrometer (ICP-MS) is a widely used instrument adopted for the fast determination of multi-elements in pharmaceutical samples including traditional herbal medicines. ICP-MS can identify and quantify trace elements with higher sensitivity due to relatively low detection limits. The concentrations of zinc, manganese, copper, cadmium and selenium in 60 traditional herbal medicines studied were determined by an ICP-MS Agilent 7500A series (Agilent Technologies, Palo Alto, CA, USA). The operating parameters and the setup information for all elements and masses are shown in Table 5 and Table 6, respectively.

### 3.7. Validation Procedure

The analytical method employed for the screening of five selected trace elements in both Chinese and Malay THM was first validated. The study consisted of linearity, precision and accuracy, limit of detection (LOD) and limit of quantification (LOQ). The Standard Reference Material, SRM 1570A, Trace Elements in Spinach from the National Institute of Standards and Technology (NIST) was chosen as the positive and negative control due to the presence of all the elements studied at specific concentrations in the SRM.

## 4. Conclusions

The concentrations of five trace elements (Mn, Zn, Cu, Se and Cd) were determined in both Chinese and Malay THM products by ICP-MS. The ranges of elemental concentrations were found to vary widely. Our results indicate that Chinese THM dominated with regard to the content of all elements studied *i.e.*, Mn, Cu, Zn, Se and Cd. The concentrations of Mn, Cu, Zn and Se present in both Chinese and Malay THMs appear to be essential, except for cadmium. Several possibilities were proposed as contributors to the presence of trace elements in both THM samples. The results show that the analytical method used in this work was applicable for the determination of Se, Cu, Mn, Zn and Cd content in the herbal medicines matrix.

## Figures and Tables

**Table 1:** Analysis of SRM1570A spinach leaves as standard reference material (μg g^−1^, *n* = 7).

Trace element	Certified concentration	Measured concentration	Recovery (%)
Zinc	82 ± 3	71 ± 2	86.4
Manganese	75.9 ± 0.6	72.5 ± 0.3	95.6
Copper	12.2 ± 0.6	11.5 ± 0.3	86.5
Cadmium	2.89 ± 0.07	2.47 ± 0.02	85.3
Selenium	0.117 ± 0.009	0.116 ± 0.003	98.9

**Table 2:** Coefficient of variation (CV) of the measurements (*n* = 12).

Trace element	Coefficient of variation (%)	

	Intraday precision	Interday precision
Zinc	2.41	1.35
Manganese	0.54	0.74
Copper	2.24	1.65
Cadmium	0.86	1.11
Selenium	2.65	3.58

**Table 3:** Limit of detection (LOD) and limit of quantitation (LOQ) of all elements studied with (*n* = 10); ng L^−1^.

Element	Copper	Zinc	Manganese	Selenium	Cadmium
LOD	0.38	0.05	0.28	0.21	0.03
LOQ	1.15	0.15	0.84	0.64	0.10

**Table 4:** Microwave digestion operating conditions.

Step	Power (W)	% max	Time (min) to raise temperature	Temperature (°C)	Running time (min)
1	400	100	15	200	5
2	400	100	1	210	5
3	400	100	1	220	5

**Table 5:** ICP-MS operating conditions for the ICP-MS equipped with an octopole reaction system.

ICP-MS System	Parameter
RF Power	1550 watts
RF Matching	1.55 V
Reflected Power	0 W
Sample Uptake Time	30 sec
Sample Uptake Rate	0.4 r sec^−1^
Sample Depth	5.0–5.5 mm
Coolant Argon Flow Rate	15 L min^−1^
Carrier Gas Flow Rate	1.2 L min^−1^
Auxiliary gas flow rate	0.9 L min^−1^
Water RF/TP Flow Rate	2.4 L min^−1^
Water RF/TP Temperature	20 °C

**Table 6:** Setup information for elements and masses.

Element	Mass	Mode	Integration Time (sec per point)
Zn	66	He	0.10
Mg	24	He	0.05
Cu	63	He	0.10
Cd	111	No gas	1.00
Se	78	He/H_2_	5.00
